# ReMindCare App for Early Psychosis: Pragmatic Real World Intervention and Usability Study

**DOI:** 10.2196/22997

**Published:** 2020-11-06

**Authors:** Lucia Bonet, John Torous, David Arce, Ignacio Blanquer, Julio Sanjuan

**Affiliations:** 1 Department of Mental Health Sanitary Research Institute of Valencia University Clinic Hospital of Valencia Valencia Spain; 2 Faculty of Medicine and Odontology University of Valencia Valencia Spain; 3 Division of Digital Psychiatry Department of Psychiatry Beth Israel Deaconess Medical Center, Harvard Medical School Boston, MA United States; 4 Institute of Instrumentation for Molecular Imaging Joint Centre of the Spanish National Research Council and Universitat Politècnica de València Valencia Spain; 5 Centre of Biomedical Investigation in Mental Health Spanish Government Carlos III Health Institute Madrid Spain

**Keywords:** app, clinical practice, mental health, psychosis, real-world intervention, telemedicine

## Abstract

**Background:**

eHealth interventions are widely used in clinical trials and increasingly in care settings as well; however, their efficacy in real-world contexts remains unknown. ReMindCare is a smartphone app that has been systematically implemented in a first episode of psychosis program (FEPP) for patients with early psychosis since 2018.

**Objective:**

The objective of this study was to assess the efficacy of ReMindCare after 19 months of use in the clinic and varying use by individual patients.

**Methods:**

The integration of the ReMindCare app into the FEPP started in October 2018. Patients with early psychosis self-selected to the app (ReMindCare group) or treatment as usual (TAU group). The outcome variables considered were adherence to the intervention and number of relapses, hospital admissions, and visits to urgent care units. Data from 90 patients with early psychosis were analyzed: 59 in the ReMindCare group and 31 in the TAU group. The mean age of the sample was 32.8 (SD 9.4) years, 73% (66/90) were males, 91% (83/90) were White, and 81% (74/90) were single.

**Results:**

Significant differences between the ReMindCare and TAU groups were found in the number of relapses, hospitalizations, and visits to urgent care units, with each showing benefits for the app. Only 20% (12/59) of patients from the ReMindCare group had a relapse, while 58% (18/31) of the TAU patients had one or more relapses (χ^2^=13.7, *P*=.001). Moreover, ReMindCare patients had fewer visits to urgent care units (χ^2^=7.4, *P*=.006) and fewer hospitalizations than TAU patients (χ^2^=4.6, *P*=.03). The mean of days using the app was 352.2 (SD 191.2; min/max: 18-594), and the mean of engagement was 84.5 (SD 16.04).

**Conclusions:**

To our knowledge, this is the first eHealth intervention that has preliminarily proven its benefits in the real-world treatment of patients with early psychosis.

**International Registered Report Identifier (IRRID):**

RR2-10.1111/eip.12960

## Introduction

High interest in eHealth services and now digital and mobile health has been noted in many recent studies among patients with psychotic disorder diagnoses [1,2]. With COVID-19, this interest in digital health has surged, and the need to expand access to care through smartphones has become patent. Smartphone apps have been proposed as tools to mitigate social isolation, lack of access to care, and other triggers caused by the pandemic [3-5]. Researchers have already demonstrated that access to and use of technology among people with psychosis is nearly equivalent to that in the general population [6-8], but less is known about the actual efficacy of apps in care.

Apps have already seen growth in care for patients with early course psychosis. Many studies are using real-time ecological momentary assessment (EMA) surveys to monitor symptoms and experiences and identify early indicators of relapse [9]. Beyond relapse prediction, these EMA data can offer novel information on the longitudinal health status of patients, which could improve treatment and shared decision making between patient and physician [10]. Finally, eHealth services may be a major resource to enhance the benefits of the first episode of psychosis programs (FEPPs) for early psychosis, which can foster recovery [11] and reduce the risk of hospitalization and relapse [12,13].

Specific apps targeting schizophrenia have already been created and offer promising results. Examples of these innovative interventions are the Actissist [14] and the ExPRESS [15] interventions, which demonstrated potential in improving the quality of treatment of patients with early psychosis. Another example is the CrossCheck app [16], which demonstrated potential for identifying and dismantling dysfunctional beliefs that contribute to maintenance and distress associated with psychotic symptoms. Despite the widespread use of these eHealth interventions and high rates of efficacy reported in clinical trials, the efficiency and actual efficacy of these interventions in real-world clinical practice remains unknown [17].

One reason for the lack of initial success of health apps in clinical settings is lack of engagement. Often engagement in academic studies does not translate into real-world use [18,19]. Indeed, some studies found a negative correlation between the time spent using eHealth apps and the engagement of patients [20,21]. In addition, many clinicians expressed their concern that if these systems integrate seamlessly with clinical workflow, they will result in an increase in the clinicians’ workload [22,23], which might affect their engagement with the app.

Other concerns have also limited efforts to integrate these apps into care settings. In our previous study [8], we found that 20% to 23% of patients felt anxious, suspicious, or paranoid concerning the internet, and almost 25% of patients perceived that use of the internet was directly related to one of their relapses. In addition, some studies indicated that excessive eHealth communications could be regarded as intrusive or irritating [24,25] or could increase worries about illness [25]. These potential harms of eHealth interventions must also be taken into consideration.

Considering these factors, it is clear that eHealth interventions shown to be feasible must now be assessed for effectiveness, efficacy, and efficiency [26] in real-world settings. With this objective in mind and to improve the daily treatment of patients with psychosis, we designed the ReMindCare app. The protocol followed for the design process and implementation of the app is published elsewhere [27]. In this protocol, we introduced ReMindCare as a smartphone app plus a clinician dashboard, developed to be implemented in a FEPP for patients with early psychosis.

To the best of our knowledge, ReMindCare is the first eHealth intervention for patients with early psychosis that has been systematically integrated into daily clinical practice, finally filling the gap between research and clinical practice [2,17].

The aim of this study was to assess the efficacy and clinical outcomes of the use of the app after 19 months in terms of adherence to ReMindCare, relapse prevention, hospital admissions, and visits to urgent care units compared with treatment as usual (TAU) without the app.

## Methods

### Study Setting

The app was systematically integrated into the daily clinical workflow in a FEPP at the University Clinic Hospital of Valencia, Spain. This FEPP started in 2010 with the objective of improving early detection, evaluation, and personalization of treatment. It covers a total of 330,000 inhabitants included in Area 5 of Valencia city. The incidence of novel psychotic disorders in this area has gradually increased during the 10 years since the program started. Currently, the FEPP in the clinic hospital has a mean of 30 to 35 new patients with psychosis per year.

The implementation of the ReMindCare app into the FEPP and into clinical practice started in October 2018 and is still in use today. In this study, we present the results from the first 19 months of use of the app.

Neither patients nor physicians received any remuneration or compensation for participating in the program or using the app. The use of the app was offered as an extra free service to the patients in the program.

### Participants

#### Recruitment and Enrollment

The patient’s psychiatrist of reference offered the use of the ReMindCare app to every outpatient from the FEPP who met the criteria for inclusion. Once patients enrolled in the study, they were encouraged to use the app as long as they remained in the program (maximum period of 5 years). To use the app, all patients signed an informed consent form and completed baseline assessments.

#### Eligibility Criteria

To be considered for this intervention, patients met the following criteria: (1) diagnosis of psychotic disorder following DSM-5 (*Diagnostic and Statistical Manual of Mental Disorders, 5th Edition*) criteria, interview conducted by a licensed clinician, (2) aged between 17 and 65 years, (3) smartphone ownership with an internet connection that allows for the proper installation and functioning of the app, and (4) less than 5 years of illness duration. However, it must be stated that some patients remained in the program for more than 5 years. These patients remained in the FEPP to prevent potential relapses, as they experienced severe fluctuations in their symptoms.

Criteria for exclusion were (1) lack of ability to use and master a mobile device and the internet, (2) refusal to sign an informed consent form, and (3) level of Spanish or English not fluent enough to maintain a conversation or understand the app questionnaires.

### Intervention

#### ReMindCare App

**Figure 1:**
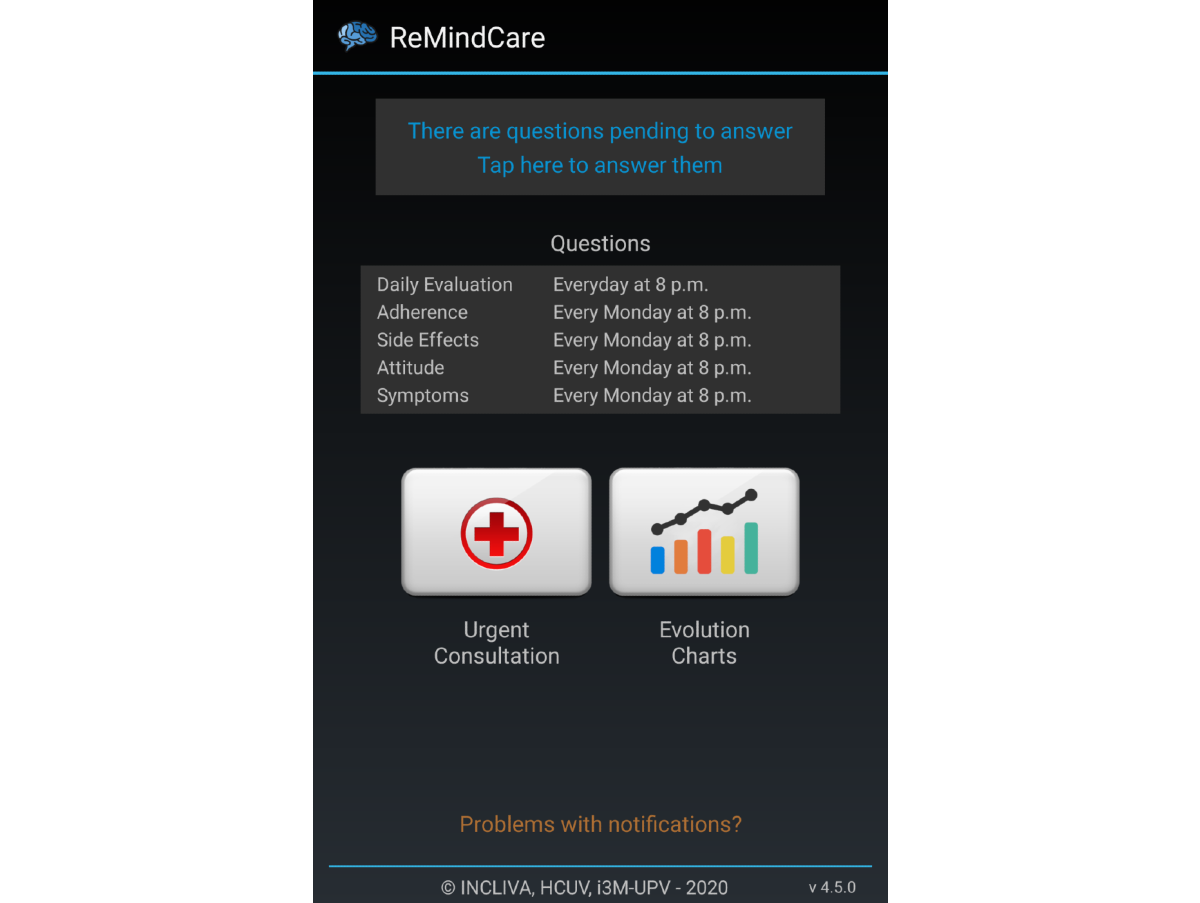
Screenshot of the ReMindCare app home screen.

ReMindCare is a free and user-friendly app that conducts daily evaluations of the health status of patients with early psychosis by offering quick questionnaires (Figure 1).

**Figure 2:**
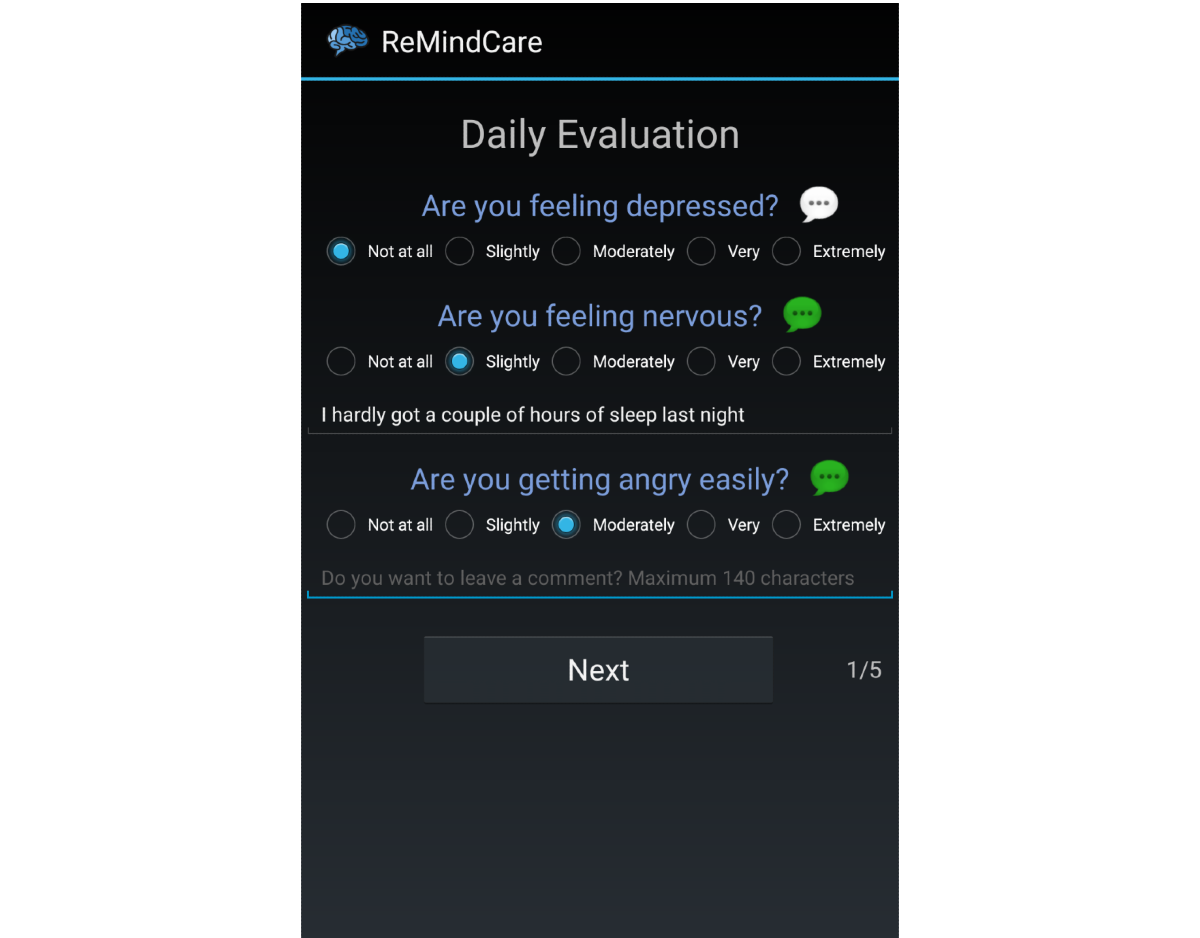
Screenshot of the ReMindCare daily questionnaire.

Two types of questionnaires were included:

- Daily questionnaires: 3 daily questions assessing levels of anxiety, sadness, and irritability (Figure 2)
- Weekly questionnaires: 18 weekly questions aimed at assessing adherence to medication (1), the presence of side effects from antipsychotic medication intake (5), the attitude toward medication intake (3), and the presence of prodromal psychosis symptoms (9)

In addition, the app offered preset alerts in case of low engagement or abrupt changes in survey responses. Low engagement alerts were set off if patients did not respond to the surveys for 7 days or more, while abrupt changes were considered when there was a difference of 2 points (Likert scale 1 to 5) or more between each question in the last 2 surveys answered. These alerts notified physicians by email and were also displayed in the profile of the patient on the app’s website portal.

All data captured by the app were accessible for physicians on a password-protected dashboard. Moreover, physicians could download a summary pdf of these data from the dashboard and attach it to the electronic clinical record of the patient in the hospital database.

The app is available in 3 languages (Spanish, English, and Catalán), although we are open to developing new language versions of the app. Our aim is to extend the use of the app to other countries, and adaptation of the app to different languages would be necessary to ensure patient engagement. Further information about the design process of the app and its characteristics can be found in the ReMindCare app study protocol [27].

Patients who used the app (ReMindCare group) did not experience any changes in their usual clinical appointments.

#### Treatment as Usual

The TAU group comprised patients who met the criteria but rejected using the app. In this group of patients, 42% (13/31) were patients with low adherence to treatment, 26% (8/31) did not perceive any benefit from using the app, and 26% (8/31) were suspicious about technology and their privacy. Additionally, 6% (2/31) were included in this group because they only used the app for 2 days. These patients continued with their usual psychiatric treatment at the FEPP and were not adversely affected by their rejection of participation.

### Procedure

Once patients enrolled in the FEPP, after an interview with their psychiatrist of reference, they were asked to complete some baseline assessments. Subsequently, they were offered the use of the ReMindCare app. The ReMindCare app was described as an extra tool developed by the FEPP that could help them manage their symptoms and help clinicians better understand their illness evolution. The main characteristics of the app were listed. After receiving this information, patients decided whether they were willing to use the app. If they were not interested, they were placed in the TAU group. If patients were interested, they were informed in more detail by an expert clinician about the installation process, characteristics of the app, and ethics and data privacy information.

Patients could use the ReMindCare app to contact their psychiatrist of reference directly in case of symptoms worsening by using the urgent consultation request tab on the home screen of the app. If they clicked the urgent consultation request, their clinician would contact them by phone within 48 hours (patients who did not use the app could call the department of psychiatry at the hospital and be referred to their psychiatrist or attend an urgent care unit). In addition, clinicians contacted patients by phone in response to preset alarms. As a result of these phone calls and the information that patients provided to the clinician, urgent care visits could be scheduled if necessary. With these services, we aimed to improve the detection of early psychotic symptoms and reduce the visits to urgent care units at the hospital, as these prodromal symptoms will be primarily treated by a phone call or in the outpatient services. If patients did not make an urgent consultation request and no preset alarms were set off, they continued with their scheduled clinical appointments.

Furthermore, the use of the ReMindCare app changed the dynamics of the clinical appointment at the outpatient services. Once patients arrived at the clinical appointment, physicians accessed their profile on the ReMindCare’s physician dashboard and used the information provided for patients to guide them through the interview. Clinicians used shared decision making with patients and discussed their responses.

### Data Collection and Measures

#### Baseline

After patients were enrolled in the FEPP, the following data were collected:

- Sociodemographic information: age, gender, country, ethnicity, marital status, education level, employment status, and cohabitation
- Clinical information: antipsychotic medication, injectable medication, length of illness, associated illnesses, suicidal attempts, Clinical Global Impression Severity of Illness scale (CGI-SI) [28], Global Assessment of Functioning (GAF) [29], Positive and Negative Syndrome Scale (PANSS) [30], Premorbid Adjustment Scale (PAS) [31], date discharged from FEPP

#### Outcome Measures

- Efficacy: number of relapses, number of visits to the hospital urgent care units, and number of hospital admissions in the ReMindCare group compared with the TAU group
- Feasibility: number of patients who agreed to use the app compared with the patients who did not use it (TAU)
- Compliance and engagement: number of times patients answered the questionnaires when presented and number of months using the app, patients dropouts, plus number of urgent consultation requests

### Data Analysis

Data were analyzed with the statistical program SPSS Statistics version 22 (IBM Corp). The cohort was divided into two groups: ReMindCare group patients agreed to use the app and used it for at least 1 month; the TAU group patients did not use the app or used it for less than 1 month. To consider that patients in the ReMindCare group had a relapse while using the app, patients had to be actively using the app. Relapses of patients who did not use the app for more than 2 months were not considered as relapses while using the app. Descriptive statistics (mean, standard deviation, frequency, and percentage) were determined, and chi-square test analysis was performed to compare the differences between the ReMindCare group and the TAU group.

### Ethics, Data Privacy, and Participant Safety

The ReMindCare app project received approval from the research ethics committee of the faculty of medicine at the University of Valencia and from the research ethics committee of the Sanitary Research Institute of the University Clinic Hospital of Valencia, Spain.

To protect the data sent by patients, communications to the platform were encrypted with a transport layer security certificate from the Generalitat Valenciana and were sent through the https protocol. The hospital infrastructure is protected through a reverse proxy, which enhances security by establishing a single access point to it and hiding all inner infrastructures. Moreover, the integration of the app into the hospital systems was subjected to Organic Law 3/2018: protection of personal data and digital rights guarantee, December 5th, the Spanish organic law adaptation of the General Data Protection Regulation.

## Results

Data from 90 patients were analyzed: 59 used or are using the app (ReMindCare group) and 31 did not agree to use the app (TAU group). Characteristics of both groups are displayed in Tables 1 and 2.

**Table 1:** Sociodemographic data.

Characteristic		Total	RC^a^ group	TAU^b^	χ^2^ (*P* value)
**Age in years, mean (SD)**		32.8 (9.4)	32.1 (1.2)	34.3 (1.7)	1.5 (.57)
	24 and younger, n (%)	19 (21)	12 (20)	7 (23)	—^c^
	25-44, n (%)	58 (64)	40 (68)	18 (58)	—
	45 and older, n (%)	13 (14)	7 (12)	6 (19)	—
Gender (male), n (%)		66 (73)	40 (68)	25 (81)	1.7 (.19)
Native country (Spain), n (%)		79 (87)	48 (81)	30 (97)	4.2 (.04)
Race (White), n (%)		83 (91)	51 (86)	31 (100)	4.6 (.33)
**Marital status, n (%)**		—	—	—	5.2 (.16)
	Single	74 (81)	50 (85)	23 (74)	—
	Married	11 (12)	5 (9)	6 (19)	—
	Other	85 (7)	4 (7)	2 (7)	—
**Educational level, n (%)**		—	—	—	5.9 (.05)
	Primary	2 (2)	0 (0)	2 (7)	—
	Secondary	45 (50)	27 (46)	18 (58)	—
	College or higher	43 (48)	32 (54)	11 (36)	—
**Employment status, n (%)**		—	—	—	5.6 (.24)
	Employed	29 (32)	16 (27)	13 (42)	—
	Student	21 (23)	16 (27)	4 (13)	—
	Not employed	38 (42)	25 (42)	13 (42)	—
	Unable to work	3 (3)	2 (3)	1 (3)	—
**Cohabitation, n (%)**		—	—	—	2.3 (.51)
	Alone	6 (7)	3 (5)	3 (10)	—
	Family_birth	60 (66)	39 (66)	20 (65)	—
	Family_own	11 (12)	6 (10)	5 (16)	—
	Other	14 (15)	11 (19)	3 (10)	—

^a^RC: ReMindCare.

^b^TAU: treatment as usual.

^c^not applicable.

**Table 2:** Baseline clinical information.

Characteristics				Total		RC^a^ group		TAU^b^		χ^2^ (*P* value)
Injectable medication, n (%)				18 (20)		8 (14)		10 (32)		4.4 (.03)
**Length of illness in years, mean (SD)**				10.5 (2.8)		3.9 (0.4)		5.7 (0.5)		12.3 (.002)
	0-1, n (%)			13 (14)		13 (22)		0 (0)		—^c^
	2-5, n (%)			43 (48)		30 (51)		13 (42)		—
	More than 6, n (%)			34 (38)		16 (27)		18 (58)		—
Associated illnesses, n (%)				29 (32)		18 (31)		11 (36)		0.2 (.63)
Suicidal attempts, n (%)				16 (18)		12 (22)		3 (10)		2.1 (.15)
**CGI-SI^d^, mean (SD)**				4.2 (0.9)		4.1 (0.1)		4.4 (0.1)		2.7 (.26)
	Mild (1-3), n (%)			13 (16)		10 (19)		3 (11)		—
	Moderate (4-5), n (%)			66 (83)		42 (81)		24 (86)		—
	Severe (>5), n (%)			1 (1)		0 (0)		1 (4)		—
**GAF^e^, mean (SD)**				60.7 (10.9)		61.3 (1.7)		59.8 (1.7)		1.3 (.52)
	Mild (71-100), n (%)			8 (10)		4 (8)		4 (14)		—
	Moderate (51-70), n (%)			51 (65)		35 (69)		16 (57)		—
	Severe (<50), n (%)			20 (25)		12 (24)		8 (29)		—
**PANSS^f^, mean (SD)**				65.9 (18.8)		64.5 (2.2)		68.7 (4.6)		52.1 (.28)
	Positive			18.4 (6.5)		18.7 (5.8)		18.7 (6.8)		23.9 (.58)
	**Negative**			18.9 (6.9)		15.4 (5.1)		17.9 (9.3)		28.2 (.17)
		N5. Difficulty in abstract thinking	2.3 (1.3)		2.0 (0.2)		2.8 (1.5)		12.8 (.03)	
		N6. Lack of spontaneity and flow conversation	1.7 (1.3)		1.6 (1.1)		1.9 (1.7)		12.9 (.02)	
	**General**			32.3 (8.2)		66.1 (14.7)		70.5 (22.2)		32.2 (.41)
		G5. Mannerism and posturing	1.1 (0.7)		1.1 (0.4)		1.3 (0.7)		9.9 (.01)	
PAS^g^, mean (SD)				10.5 (2.8)		10.7 (0.5)		10.14 (0.6)		9.1 (.70)
**Relapses_Baseline, n (%)**				—		—		—		4.3 (.12)
	0			53 (59)		38 (64)		15 (48)		—
	1			21 (23)		14 (24)		7 (23)		—
	≥2			16 (18)		7 (12)		9 (29)		—
**UCU^h^ visits_Baseline, n (%)**				—		—		—		0.9 (.61)
	0			26 (29)		19 (32)		7 (23)		—
	1			36 (40)		23 (39)		13 (42)		—
	≥2			28 (31)		17 (29)		11 (36)		—
**Hospitalizations_Baseline, n (%)**				—		—		—		4.6 (.10)
	0			19 (21)		16 (27)		3 (10)		—
	1			50 (56)		32 (54)		18 (58)		—
	≥2			21 (23)		11 (19)		10 (32)		—

^a^RC: ReMindCare.

^b^TAU: treatment as usual.

^c^not applicable.

^d^CGI-SI: Clinical Global Impression Severity of Illness scale

^e^GAF: Global Assessment of Functioning.

^f^PANSS: Positive and Negative Syndrome Scale.

^g^PAS: Premorbid Adjustment Scale.

^h^UCU: urgent care units.

### Sociodemographic Analysis

The mean age of the sample was 32.8 (SD 9.4) years, 73% (66/90) were males, 91% (83/90) were White, and 81% (74/90) were single. No significant differences were found between the ReMindCare and TAU groups in any of the sociodemographic information analyzed except for the native country. We found that nearly every immigrant considered for inclusion agreed to use the app (ReMindCare group 19% [10/11], TAU group 3% [1/11]; χ^2^=4.2, *P*=.04). Further information regarding sociodemographic analysis of the data is displayed in Table 1.

### Baseline Clinical Analysis

Significant differences were found between the ReMindCare group and TAU group in some clinical factors. With regard to injectable medication, 32% (10/31) of TAU patients were taking injectable medication, while only 14% (8/59) of the ReMindCare took it (χ^2^=4.4, *P*=.04). Every new patient in the FEPP (length of illness: 0-1 year) agreed to use the app (13/90, 22%), and 58% (18/31) of the TAU group had their illness for 6 or more years (χ^2^=12.3, *P*=.002). Moreover, the TAU patients showed higher scores on the PANSS N5 and N6 negative subscales and G5 in the general subscales (χ^2^=12.8, *P*=.03; χ^2^=12.9, *P*=.02; χ^2^=9.9, *P*=.01, respectively).

Considering medication, 20% (18/90) of patients were taking injectable medications, 32% (29/90) of the patients suffered from another illness, and 18% (17/90) had a prior suicidal attempt. The mean of the CGI-SI was 4.2 (SD 0.9), the GAF mean=60.7 (SD 10.9), PANSS mean 65.9 (SD 18.8), and PAS mean 10.5 (SD 2.8). Finally, 12% (11/90) of patients were discharged from the FEPP. No significant differences were found between the groups in any of these factors. Moreover, no significant differences were found between the ReMindCare group and TAU group in terms of the number of relapses (χ^2^=4.3, *P*=.12), visits to urgent care units (χ^2^=0.9, *P*=.61), or the number of hospitalizations (χ^2^=4.6, *P*=.10) at baseline. Further clinical information is available in Table 2.

### ReMindCare Outcomes

The mean of days using the app was 352.2 (SD 191.2), which corresponds to 11.6 months. The mean of compliance was 84.5 (16.04), and 61.1% of the ReMindCare group had a compliance rate between 85% and 100%.

Of the 59 ReMindCare patients, 31% (18/59) requested an urgent consultation, 20% (12/59) had a relapse while using the app, and 8% (2/59) developed a delusion involving the app and the research group.

After 19 months of intervention, 63% (37/59) of patients continued using the app, while 12% (7/59) stopped using the app because they were discharged from the FEPP and 25% (15/59) opted to stop using ReMindCare. Reasons for discontinuation: 33% (5/15) of patients felt suspicious about technology (among these patients, 4 had a relapse while using the app); 40% (6/15) perceived the app as boring and did not perceive any benefit; and 27% (4/15) of patients left treatment and did not continue in the program. This information is shown in Table 3.

**Table 3:** Use of ReMindCare.

Characteristic			RC^a^ group (n=59)	Min-max
Days using app, mean (SD)			352.2 (191.2)	18-594
Months using app, mean (SD)			11.6 (6.5)	0-19
**Engagement, mean (SD)**			84.5 (16.0)	42-100
	85%-100%, n (%)		36 (61)	—^b^
UCU^c^, n (%)			18 (31)	—
Relapses using app, n (%)			12 (20)	—
Relapses related to app, n (%)			2 (8)	—
**Status of use after 19 months, n (%)**				
	Patients using app		37 (63)	—
	**Patients not using app**		22 (37)	—
		Discharged from FEPP^d^	7 (32)	—
		Dropouts	15 (68)	—

^a^RC: ReMindCare.

^b^not applicable.

^c^UCU: urgent care units.

^d^FEPP: first episode of psychosis program.

With regard to the clinical outcomes, after 19 months of ReMindCare’s integration into the clinical workflow, only 20% (12/59) of patients from the ReMindCare group had a relapse, while 58% (18/31) of TAU patients had one or more relapses (χ^2^=13.7, *P*=.001). Moreover, ReMindCare patients had fewer visits to urgent care units (χ^2^=7.4, *P*=.006) and fewer hospitalizations than TAU patients (χ^2^=4.6, *P*=.03). Information regarding these clinical outcomes is displayed in Table 4.

**Table 4:** Clinical outcomes after 19 months of the ReMindCare intervention.

Characteristic		Total, n (%)	RC^a^ group, n (%)	TAU^b^, n (%)	χ^2^ (*P* value)
**Relapses**		—^c^	—	—	13.7 (.001)
	0	60 (67)	47 (80)	13 (42)	—
	1	29 (32)	12 (20)	17 (55)	—
	≥2	1 (1)	0 (0)	1 (3)	—
UCU^d^ visits		20 (22)	8 (14)	12 (39)	7.4 (.006)
Hospitalizations		9 (10)	3 (5)	6 (19)	4.6 (.03)

^a^RC: ReMindCare.

^b^TAU: treatment as usual.

^c^not applicable.

^d^UCU: urgent care units.

## Discussion

### Principal Findings

The results obtained from these analyses of the first 19 months of ReMindCare use highlight the potential benefits of this eHealth intervention for patients with early psychosis. Patients who used the app not only had fewer relapses than the TAU group, but they also had fewer visits to the urgent care unit and fewer hospitalizations.

Results related to the efficacy of the app are in line with previous results obtained in clinical trials [14-16]. However, as far as we know, this is the first study to identify the benefits of the use of an app as a tool systematically integrated into daily clinical practice in a FEPP.

With regard to the feasibility of the app, no significant differences were found between the ReMindCare group and the TAU group in terms of sociodemographic characteristics except for native country. The feasibility of this intervention aligns with the results obtained in our previous study [8], where we found no differences in terms of sociodemographic characteristics and interest in using eHealth interventions.

With regard to the clinical characteristics of the samples and their impact on the effect of ReMindCare, there were some differences between groups. We found that patients who did not use the app were more likely to be taking injectable medication, have a longer history of illness, and have higher scores on the PANSS N5 and N6 negative subscales and G5 in the general subscales. These results might suggest that the use of ReMindCare was not indicated for chronic patients. However, we did not find differences in other clinical scales such as the CGI-SI, GAF, and PAS scales or even on the PANSS total scale. More importantly, we did not find any differences between groups in terms of baseline relapses, hospitalizations, or visits to urgent care units.

These results are in line with the ones we obtained in our previous study [8], where we found that interest in using eHealth apps was equivalent between chronic and early psychosis patients. In this regard, we suggest that differences obtained in terms of the clinical characteristics of the patients could be more related to the history of treatment than to clinical characteristics. As we found, every new patient who joined the FEPP (length of illness less than 1 year) was interested in using the app (22% of users), while patients who had a longer history of treatment (length of illness more than 6 years) were more likely to reject its use (58% of TAU group). This could highlight the relevance of introducing these new technologies at the very beginning of treatment so early psychosis patients consider these apps to be just another tool included in their daily clinical treatment and not an extra service, especially since our results suggested that use of the app had a significant impact in improving the course of the illness.

Finally, with regard to compliance and engagement with the app, we found that 61% of patients had compliance rates between 85% to 100%. Rates of engagement were also high, as 63% of patients still use the app after almost 1 year. These results of compliance and long-term engagement are contrary to previous studies [20,21] and suggest that the use of an app in a long-term approach is feasible and beneficial.

However, we would like to highlight that 20% of patients had a relapse while using the app and 8% developed a delusion involving the use of the app and the research group. These negative results should be cautiously considered.

Technology could be a major resource to improve the quality of treatments, but as we found in a previous study [8], it can also play an important role as a trigger for psychotic symptoms. In this regard, in a 3-case study in 2011 conducted by Nitzan et al [32], they stated that the use of the internet and computers might contribute to a gradual break with reality and development of psychotic symptoms. They suggested that given that patients with psychotic diagnoses have greater difficulties in filtering and understanding signals and symbols, they are also more likely to misinterpret digital messages. However, no specific studies regarding the potential harms of the use of new technologies have been undertaken until the present.

In our study, we found that the ReMindCare app was related to beneficial clinical effects for the vast majority of patients who used it. However, despite the general positive effects found in this study, there are still some barriers and negative effects that must be taken into consideration. The main barrier found in our study relates to the 34% of the approached patients who did not want to use the app and who also tended to be the more chronic patients. Moreover, the main negative effect we found related to the 8% of patients who developed a delusion involving the app. As a result, we would like to point out that this app is not a panacea to prevent relapses. However, it is clear that the app positively affected the course of the illness, as only 5% of those who relapsed required hospitalization compared with 19% of patients who relapsed in the TAU group.

### Limitations and Strengths

There were some limitations that must be taken into consideration. First, not every outpatient from the FEPP was eligible for inclusion, as some patients did not have their own smartphone with an internet connection or did not have the ability to use the app or understand it due to language barriers. Developing strategies to prevent digital exclusion should be a priority to ensure that every patient could benefit from these technologies [33]. Second, as a real-world study, this study was not randomized. Despite the groups not differing in the vast majority of clinical or demographic characteristics, there were some factors such as personality that could influence our results.

The main strength of our study was the fact that ReMindCare is the first app that has been systematically integrated into the clinical FEPP workflow. To our knowledge, there are no previous studies that used an app as a tool to improve the daily treatment of patients with early psychosis. All the studies we found were conducted in academic research settings that did not emulate real-world environments [17,34].

Another strength is in regard to the development of the ReMindCare app. First, it was based on two previous studies [2,8] and co-designed with patients [27]. Second, we conducted a pilot study and focus groups to ensure the involvement of both patients and care providers [27] in the design and improvement process of the app.

Finally, we would like to highlight the long-term approach of this intervention. As stated before, ReMindCare is now integrated into clinical practice and it was used for 19 months. These results align with previous studies [16] that found that people with psychosis have the abilities and interest required to engage in long-term eHealth interventions.

### Implications for the Future

As a result of these analyses, we highlighted the benefits that the use of ReMindCare app produced on early psychosis patients in a FEPP. Our aim is to continue improving the app in response to the needs and suggestions provided by patients and clinicians. As Ross et al [22] claimed in their meta-review, in order to ensure the use of these eHealth technologies over time, there are three challenges that should be overcome. First, the apps must be able to adapt to the characteristics of the environment and patients. Second, the apps should be easy to use. Third, the apps should be integrated into clinical practice, adjusting the characteristics of the app in order to ensure it is user-friendly and efficient for patients and clinicians. It is our aim to address these issues to maintain the positive results obtained in this study.

However, we would like to point out a major issue that must guide future eHealth interventions. As stated before, 8% of patients developed a delusion related to the use of the app, 25% of patients deliberately stopped using the app, and 34% of patients approached did not want to use the app in the first place. These results suggest that there are still significant numbers of patients not willing to use eHealth interventions, and there are some patients who could be adversely affected by the use of these technologies. Studying the characteristics of these patients should guide future research in order to ensure that the use of digital technologies only provides benefits to the patients [8].

Finally, we would like to underline that given the exceptional situation that the world is facing at the moment with COVID-19 and in order to address the requirements of interventions that could improve the telematic treatment of patients and prevention of hospital collapses [4,35], ReMindCare could be used as an effective and efficient tool. Since quarantining in Spain began March 13, 2020, patients have not been permitted to come in person to their clinical appointments and have received their clinical evaluations by phone. Since that moment, the use of ReMindCare has been extremely useful to improve the evaluation and adherence of early psychosis patients. However, future analysis will be conducted in regard to this aspect.

As the conclusion of this study, we would like to point out that, to the best of our knowledge, ReMindCare is not only the first app to be integrated into the clinical practice, it is the first eHealth intervention with evidence that it improves the outcomes of early psychosis patients in a real-world care setting.

## Abbreviations

CGI-SI: Clinical Global Impression Scale-Severity Illness
DSM-5: Diagnostic and Statistical Manual of Mental Disorders, 5th Edition
EMA: ecological momentary assessment
FEPP: first episode of psychosis program
GAF: Global Assessment of Functioning
PANSS: Positive and Negative Syndrome Scale
PAS: Premorbid Adjustment Scale
TAU: treatment as usual

## References

[1] Firth J, Cotter J, Torous J, Bucci S, Firth JA, Yung AR (2015). Mobile phone ownership and endorsement of “mHealth” among people with psychosis: a meta-analysis of cross-sectional studies. Schizophr Bull.

[2] Bonet L, Izquierdo C, Escartí MJ, Sancho JV, Arce D, Blanquer I, Sanjuan J (2017). Use of mobile technologies in patients with psychosis: a systematic review. Rev Psiquiatr Salud Ment.

[3] Kannarkat JT, Smith NN, McLeod-Bryant SA (2020). Mobilization of telepsychiatry in response to COVID-19: moving toward 21 century access to care. Adm Policy Ment Health.

[4] Torous J, Keshavan M (2020). COVID-19, mobile health and serious mental illness. Schizophr Res.

[5] Wang C, Pan R, Wan X, Tan Y, Xu L, McIntyre R, Choo FN, Tran B, Ho R, Sharma VK, Ho C (2020). A longitudinal study on the mental health of general population during the COVID-19 epidemic in China. Brain Behav Immun.

[6] Trefflich F, Kalckreuth S, Mergl R, Rummel-Kluge C (2015). Psychiatric patients' internet use corresponds to the internet use of the general public. Psychiatry Res.

[7] Gay K, Torous J, Joseph A, Pandya A, Duckworth K (2016). Digital technology use among individuals with schizophrenia: results of an online survey. JMIR Ment Health.

[8] Bonet L, Llácer B, Hernandez-Viadel M, Arce D, Blanquer I, Cañete C, Escartí M, González-Pinto AM, Sanjuán J (2018). Differences in the use and opinions about new eHealth technologies among patients with psychosis: structured questionnaire. JMIR Ment Health.

[9] Hau YS, Kim JK, Hur J, Chang MC (2020). How about actively using telemedicine during the COVID-19 pandemic?. J Med Syst.

[10] Bucci S, Berry N, Morris R, Berry K, Haddock G, Lewis S, Edge D (2019). “They are not hard-to-reach clients. We have just got hard-to-reach services.” Staff views of digital health tools in specialist mental health services. Front Psychiatry.

[11] Arango C, Bernardo M, Bonet P, Cabrera A, Crespo-Facorro B, Cuesta MJ, González N, Parrabera S, Sanjuan J, Serrano A, Vieta E, Lennox BR, Melau M (2017). When the healthcare does not follow the evidence: the case of the lack of early intervention programs for psychosis in Spain. Rev Psiquiatr Salud Ment.

[12] Camacho E, Levin L, Torous J (2019). Smartphone apps to support coordinated specialty care for prodromal and early course schizophrenia disorders: systematic review. J Med Internet Res.

[13] Correll CU, Galling B, Pawar A, Krivko A, Bonetto C, Ruggeri M, Craig TJ, Nordentoft M, Srihari VH, Guloksuz S, Hui CLM, Chen EYH, Valencia M, Juarez F, Robinson DG, Schooler NR, Brunette MF, Mueser KT, Rosenheck RA, Marcy P, Addington J, Estroff SE, Robinson J, Penn D, Severe JB, Kane JM (2018). Comparison of early intervention services vs treatment as usual for early-phase psychosis: a systematic review, meta-analysis, and meta-regression. JAMA Psychiatry.

[14] Bucci S, Barrowclough C, Ainsworth J, Machin M, Morris R, Berry K, Emsley R, Lewis S, Edge D, Buchan I, Haddock G (2018). Actissist: proof-of-concept trial of a theory-driven digital intervention for psychosis. Schizophr Bull.

[15] Eisner E, Drake RJ, Berry N, Barrowclough C, Emsley R, Machin M, Bucci S (2019). Development and long-term acceptability of ExPRESS, a mobile phone app to monitor basic symptoms and early signs of psychosis relapse. JMIR Mhealth Uhealth.

[16] Ben-Zeev D, Brian R, Wang R, Wang W, Campbell AT, Aung MSH, Merrill M, Tseng VWS, Choudhury T, Hauser M, Kane JM, Scherer EA (2017). CrossCheck: integrating self-report, behavioral sensing, and smartphone use to identify digital indicators of psychotic relapse. Psychiatr Rehabil J.

[17] Torous J, Woodyatt J, Keshavan M, Tully LM (2019). A new hope for early psychosis care: the evolving landscape of digital care tools. Br J Psychiatry.

[18] Torous J, Lipschitz J, Ng M, Firth J (2020). Dropout rates in clinical trials of smartphone apps for depressive symptoms: a systematic review and meta-analysis. J Affect Disord.

[19] Killikelly C, He Z, Reeder C, Wykes T (2017). Improving adherence to web-based and mobile technologies for people with psychosis: systematic review of new potential predictors of adherence. JMIR Mhealth Uhealth.

[20] Krzystanek M, Krysta K, Skałacka K (2017). Treatment compliance in the long-term paranoid schizophrenia telemedicine study. J Technol Behav Sci.

[21] Arnold C, Villagonzalo K, Meyer D, Farhall J, Foley F, Kyrios M, Thomas N (2019). Predicting engagement with an online psychosocial intervention for psychosis: exploring individual- and intervention-level predictors. Internet Interv.

[22] Ross J, Stevenson F, Lau R, Murray E (2016). Factors that influence the implementation of e-health: a systematic review of systematic reviews (an update). Implement Sci.

[23] Allan S, Bradstreet S, Mcleod H, Farhall J, Lambrou M, Gleeson J, Clark A, Gumley A, EMPOWER Group (2019). Developing a hypothetical implementation framework of expectations for monitoring early signs of psychosis relapse using a mobile app: qualitative study. J Med Internet Res.

[24] Palmier-Claus JE, Rogers A, Ainsworth J, Machin M, Barrowclough C, Laverty L, Barkus E, Kapur S, Wykes T, Lewis SW (2013). Integrating mobile-phone based assessment for psychosis into people's everyday lives and clinical care: a qualitative study. BMC Psychiatry.

[25] Kannisto KA, Adams CE, Koivunen M, Katajisto J, Välimäki M (2015). Feedback on SMS reminders to encourage adherence among patients taking antipsychotic medication: a cross-sectional survey nested within a randomised trial. BMJ Open.

[26] Torous JB (2018). Focusing on the future of mobile mental health and smartphone interventions. Psychiatr Serv.

[27] Bonet L, Torous J, Arce D, Blanquer I, Sanjuán J (2020). ReMindCare, an app for daily clinical practice in patients with first episode psychosis: a pragmatic real-world study protocol. Early Interv Psychiatry.

[28] Busner J, Targum S (2007). The clinical global impressions scale: applying a research tool in clinical practice. Psychiatry (Edgmont).

[29] Endicott J, Spitzer RL, Fleiss JL, Cohen J (1976). The global assessment scale. A procedure for measuring overall severity of psychiatric disturbance. Arch Gen Psychiatry.

[30] Peralta Martín V, Cuesta Zorita MJ (1994). [Validation of positive and negative symptom scale (PANSS) in a sample of Spanish schizophrenic patients]. Actas Luso Esp Neurol Psiquiatr Cienc Afines.

[31] Cannon-Spoor HE, Potkin SG, Wyatt RJ (1982). Measurement of premorbid adjustment in chronic schizophrenia. Schizophr Bull.

[32] Nitzan U, Shoshan E, Lev-Ran S, Fennig S (2011). Internet-related psychosis−a sign of the times. Isr J Psychiatry Relat Sci.

[33] Greer B, Robotham D, Simblett S, Curtis H, Griffiths H, Wykes T (2019). Digital exclusion among mental health service users: qualitative investigation. J Med Internet Res.

[34] Rus-Calafell M, Schneider S (2020). Are we there yet?! A literature review of recent digital technology advances for the treatment of early psychosis. Mhealth.

[35] Choi KR, Heilemann MV, Fauer A, Mead M (2020). A second pandemic: mental health spillover from the novel coronavirus (COVID-19). J Am Psychiatr Nurses Assoc.

